# Using Direct and Indirect Estimates for Alcohol-Attributable Mortality: A Modelling Study Using the Example of Lithuania

**DOI:** 10.1159/000529200

**Published:** 2023-02-07

**Authors:** Jürgen Rehm, Huan Jiang, Kawon Victoria Kim, Robin Room, Pol Rovira, Kevin David Shield, Alexander Tran, Shannon Lange, Mindaugas Štelemėkas

**Affiliations:** ^a^Institute for Mental Health Policy Research, Centre for Addiction and Mental Health, Toronto, Ontario, Canada; ^b^Dalla Lana School of Public Health, University of Toronto, Toronto, Ontario, Canada; ^c^World Health Organization/Pan American Health Organization Collaborating Centre, Centre for Addiction and Mental Health, Toronto, Ontario, Canada; ^d^Campbell Family Mental Health Research Institute, Centre for Addiction and Mental Health, Toronto, Ontario, Canada; ^e^Institute of Clinical Psychology and Psychotherapy and Center of Clinical Epidemiology and Longitudinal Studies (CELOS), Technische Universität Dresden, Dresden, Germany; ^f^Faculty of Medicine, Institute of Medical Science, University of Toronto, Toronto, Ontario, Canada; ^g^Department of Psychiatry, University of Toronto, Toronto, Ontario, Canada; ^h^Department of International Health Projects, Institute for Leadership and Health Management, I.M. Sechenov First Moscow State Medical University, Moscow, Russian Federation; ^i^Program on Substance Abuse, Public Health Agency of Catalonia, Program on Substance Abuse and Designated WHO CC, Public Health Agency of Catalonia, Barcelona, Spain; ^j^Centre for Alcohol Policy Research, La Trobe University, Bundoora, Victoria, Australia; ^k^Department of Public Health Sciences, Centre for Social Research on Alcohol and Drugs, Stockholm University, Stockholm, Sweden; ^l^Health Research Institute, Faculty of Public Health, Lithuanian University of Health Sciences, Kaunas, Lithuania; ^m^Department of Preventive Medicine, Faculty of Public Health, Lithuanian University of Health Sciences, Kaunas, Lithuania

**Keywords:** Alcohol use, Mortality, Causes of death, Alcohol-attributable fraction, Risk relations

## Abstract

**Introduction:**

Comparative risk assessments (CRAs) for alcohol use are based on indirect estimates of attributable harm, and usually combine country-specific exposure estimates and global risk relations derived from meta-analyses. CRAs for Eastern European countries, such as Lithuania, base their risk relations not on global risk relations, but on a large Russian cohort study. The availability of a direct estimate of alcohol-attributable mortality following the 2017 implementation of a large increase in alcohol excise taxes in Lithuania has allowed a comparison of these indirect estimates with a country-specific gold standard.

**Methods:**

A statistical modelling study compared direct (predictions based on a time-series methodology) and indirect (predictions based on an attributable-fraction methodology) estimates of alcohol-attributable mortality before and after a large increase in alcohol excise taxes in Lithuania. Specifically, Russia-specific versus global relative risks were compared against the gold standard of time-series based predictions.

**Results:**

Compared to direct estimates, indirect estimates markedly underestimated the reduction of alcohol-attributable mortality 12 months post intervention by at least 63%. While both of the indirect estimates differed markedly from the direct estimates, the Russia-specific estimates were closer to the direct estimates, primarily due to higher estimates for alcohol-attributable cardiovascular mortality.

**Discussion:**

As all indirect estimates were markedly lower than direct estimates, current overall relative risks and price elasticities should be re-evaluated. In particular, global estimates should be replaced by new regional estimates based on cohort studies.

## Introduction

Most comparative risk assessments (CRAs) for alcohol consumption use an indirect method, namely Levin's formula, to estimate population-attributable fractions (PAF) [1]. Levin's formula estimates the alcohol-attributable proportion of the burden of disease outcomes by combining the distribution of alcohol exposure with the corresponding relative risks (RRs) for these outcomes ([2]; for recent CRAs for alcohol use, see [3, 4]). In the current application of PAF methodology, the distribution of alcohol exposure is derived from country-specific estimates (for larger countries, exposure data from different regions within a country may be available), while the corresponding RRs are based largely on global (non-country-specific) estimates obtained via meta-analyses [3, 4, 5]. However, applying the same set of RRs to calculate the PAF for specific countries will likely lead to biases arising from country-specific interactions between alcohol use and other risk factors (e.g., level of poverty and economic wealth [6]), as well as from different drinking patterns [7, 8].

The only exception to the use of global RRs in estimating PAFs has occurred for European countries of the former Soviet Union (Belarus, Estonia, Latvia, Lithuania, Moldova, Russia, and Ukraine), for which Russia-specific RRs have been used in the World Health Organization's Global Status Report on Alcohol and Health or similar CRAs [4, 9, 10]. The Russia-specific RRs are based on a large cohort study that took place from 1999 to 2010 [11] and are assumed to be more appropriate for many of the European countries of the former Soviet Union, due to their similarities in levels and patterns of alcohol consumption [12, 13]. Online supplementary Table S1 (see www.karger.com/doi/10.1159/000529200 for all online suppl. material) gives a comparison of the global versus Russia-specific RRs used.

Since the dissolution of the Soviet Union in December 1991, differing economic and social changes in the former Soviet Socialist Republics led to notable changes in mortality and in drinking behaviours; in the first years after the collapse of the Soviet Union, economic and social changes mainly contributed to increases in alcohol consumption and harms [14]. This raises the question of whether the Russia-specific RRs, obtained from a cohort study that ended more than a decade ago (i.e., in 2010), still describe the risk relations between alcohol use and mortality in the countries of the former Soviet Union more accurately than the global RRs derived from meta-analyses. This question is particularly relevant for the Baltic countries (Estonia, Latvia, Lithuania), which have since joined the European Union (EU), are now part of the Schengen Area, and have all been consistently classified as high-income countries since 2012 [15]. Even though there has been increased social and economic distance from the other post-Soviet countries, the Baltic countries can still be characterized by high levels of spirits consumption and heavy episodic drinking, especially among men [9, 16].

For instance, in Lithuania rapid increases in income in the early 2000s, combined with a culture of unhealthy drinking traditions and a lack of alcohol control policies, led to detrimental effects on health, social, and economic outcomes [17, 18]. However, the recent enactment of several alcohol control policies, including a marked increase in alcohol excise taxes on March 1, 2017 [17], namely 111–112% for wine and beer, and 23% for ethyl alcohol, significantly reduced levels of alcohol use and all-cause mortality [19, 20]. The decreases in alcohol use and mortality that resulted from the 2017 alcohol excise tax increases allow for a more direct methodology to estimate the alcohol-attributable burden. It also enables a comparison of the two indirect methodologies of global versus Russia-specific RRs with the gold standard of directly estimating the effects of the taxation increase [20]. Accordingly, the aim of the present study was to assess if the mortality reductions following the policy enactment of 2017 in Lithuania are better described by the global (non-specific) RRs used in most parts of the world or by Russia-specific RRs. More generally, this study can be seen as a case example for a more general questioning of the validity of the use of indirect estimates of the (change in) the number of alcohol-related deaths, either using country-specific or global RRs.

## Materials and Methods

The impact of the increase in excise taxation on mortality was estimated using both direct and indirect methods. Comparisons between methods were made visually and descriptively by looking at the overlaps of the respective uncertainty intervals and by judging plausibility based on the literature.

### Direct Methodology

Deaths avoided were directly estimated using methodology based on interrupted time-series [21, 22]. As these procedures have been reported in detail elsewhere [20, 23], the models will only be briefly described here. The methodology compares the actual deaths after an intervention, with an estimate of the deaths which would have happened without the intervention. To estimate these deaths, it is assumed that the trends and underlying processes seen prior to the intervention would continue. Accordingly, a time-series model was established to estimate the trend before the intervention, and this trend was used to predict the number of deaths in the 12 months following the intervention. The predicted numbers of deaths were then compared to the actual numbers, with the difference representing the effectiveness of the taxation policy on alcohol-attributable deaths.

This procedure assumes that the effects of the intervention are abrupt (i.e., the effects started on the day of the intervention) and permanent (for 12 months). Given that the intervention was a tax increase, it is plausible that the taxes were passed on to the consumers [24], and that these increased prices impacted the purchases of the consumers [25]. Further detail on the calculations used can be found in [20].

As indicated above, we treated these direct estimates as the gold standard as they involved the use of the least number of assumptions, by comparing a given trend with the actual number of deaths in a country, with no assumptions on RRs or on elasticities needed (see [20] for details). These direct estimates were then compared with four indirect estimates all based on attributable fraction: (a) the first on alcohol per capita consumption (APC) changes and global RR; (b) the second on APC changes with Russia-specific RR; (c) the third on using taxation changes, resulting in price differences, and then applying price elasticity before combining with global RR; and (d) the fourth as the third but combining with Russia-specific RR in the last step. These will be described in detail below.

### Indirect Methodology

A Levin-based population-preventable fraction (PPF) method for alcohol use (general methodology for CRAs, see [1, 2]; specifically for PPF, see [26]) was used to estimate the number of alcohol-attributable deaths avoided by the reduction of APC using non-specific and Russian-specific RRs. This method requires data on exposure and risk relations [2]. The PPF calculations (see Formula 1) were estimated based on the factual scenario of alcohol use in 2017 before the implementation of the increased excise taxation compared to a counterfactual scenario where alcohol exposure was estimated after the implementation of excise taxation (estimates taken from [20]). APC changes due to the increase in excise taxation were either estimated via time-series methodology or via applying price elasticities based on the meta-analyses [25, 27, 28] (for details and values used, see [20]).

**Figure:**
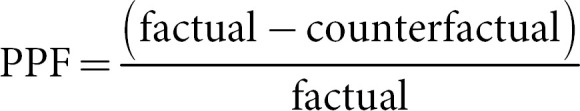

**Figure:**
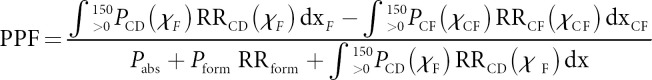

where PPF = population-preventable fraction due to taxation-based change in APC; *P* = prevalence; RR = relative risk; abs = lifetime abstainer; form = former drinker; CD = current drinker; *x* = average alcohol intake in g/day under the F = factual scenario and the CF = counterfactual scenario.

This formula was applied to each disease category causally related to alcohol use but not 100% attributable to alcohol [5]; for an overview of categories used with ICD-10 codes, see online supplementary Table S1. The exception here was cancer because the impact of alcohol use on cancer incidence and deaths takes more than 10 years and up to 20 years [29, 30, 31] to be measurable, and therefore, most changes in cancer mortality are not expected in the first year following the implementation of a policy to reduce alcohol use [30, 32]. All other disease categories, as customarily used in CRAs, were modelled as immediate effects [4], which persisted for the full year modelled.

For categories which are 100% attributable to alcohol, the alcohol PAF by definition is 1, independent of the level of consumption. To estimate changes in these disease categories (e.g., alcohol use disorders or alcohol cardiomyopathy), we followed the methodology used by Churchill and colleagues [33]; see Formula 2, where *N* is the number of deaths, *S* is the population size, RR(*x*;*k, t*) is the risk function (where *k* represents the slope of the risk function, *t* represents a minimum value for which all persons with mean daily alcohol exposure of at least *t* g/day are classified as heavy episodic drinkers (defined as drinking more than 60 g/day), and F(*x*;μ) is the gamma distribution for the mean consumption μ). Formula 2 was used to solve for *k* using derivative-free constrained optimization by linear approximations [34]. Formula 3 was used to estimate the PPF for deaths which are wholly attributable to alcohol use. To be conservative, we used an upper limit of 150 g/day.

**Figure:**
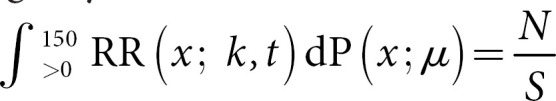

**Figure:**
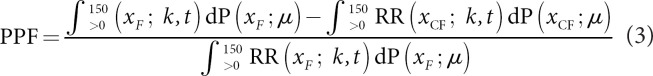

### Exposure Data

Alcohol exposure data were obtained from the Lithuanian Department of Statistics [35]. The relative alcohol use amounts by sex and age were obtained from a paper by Manthey and colleagues [12].

### Risk Relations

For the non-specific RRs, the risk relations listed in Shield and colleagues [4] were used; the Russia-specific estimates used the risk relations from the above-mentioned Russian cohort study [11], albeit with age-specific adjustments for ischemic heart disease mortality (see [2, 14]).

### Mortality Data

Monthly data on all-cause and cause-specific mortality between the years 2000 and 2019 were provided by the Lithuanian Institute of Hygiene and were age-standardized to the Lithuanian population of February 2017, the month before the excise tax change.

### Uncertainty Intervals

In order to derive the uncertainty interval (UI) for the respective estimates, Monte Carlo simulations were performed, generating 10,000 samples and using the 2.5th and 97.5th percentiles of the resulting distribution as the UI [36, 37].

### Software

All analyses were programmed in R version 4.0.1 [38].

## Results

All analyses predicted a substantial number of deaths were averted by the increase in excise taxes, but the effect sizes varied highly with non-overlapping UIs. If the direct estimate is accepted as the gold standard, the number of prevented deaths in this estimate was much larger for men than in all indirect estimates. In addition, for men and total deaths averted, the lower threshold of the 95% UI of the direct estimate was markedly higher than the upper threshold of any indirect estimate (see Table 1).

For both indirect estimates, the Russia-specific RRs estimated about twice the number of deaths averted than the global estimates did, and were closer to the gold standard, but the differences between indirect and direct estimates were considerable, even for the Russia-specific RR estimates. Tables 2 and 3 provide details of the distribution by cause of death for the higher estimate based on APC changes caused by the excise taxation increase.

For most causes of death, the numbers of deaths averted are about the same. However, for two categories the numbers are different: for cardiovascular diseases, the largest disease category in both estimates, the Russia-specific RRs estimate more than four times the number of deaths averted, and for injuries, the global RRs give a higher number. Online supplementary Table S2 compares the 12 months before and after the enactment of the taxation increase in 2017. For cardiovascular deaths, the most important category, the differences amounted to 538 less for women, 649 for men and 1,187 total. According the Russia RR estimates based on APC, the alcohol-attributable fractions would be 32%, 20%, and 25%, which would be even on the low side given other estimates, using different RR or direct estimates [29, 39]. They are in line with the direct effects, mainly for men. They also do not contradict a potential large effect of alcohol use reductions for both sexes.

## Discussion

Both indirect methodologies markedly underestimated the direct effect as estimated based on a time-series prediction model [40]. As the Russia-specific RRs were closer to the deaths predicted by the time-series analyses, the results of this study do not support giving up the current practice by switching to global RRs. However, attempts should be made to improve current indirect estimates. As recent research has indicated there are very similar impacts of comparable alcohol control policies on APC in all Baltic countries [41], indirect estimates may be derived which are not only valid for Lithuania, but for all Baltic countries.

The crucial point concerns the potential impact of alcohol on cardiovascular deaths. Most of the deaths prevented after 2017 fell into this broad category [20], raising the question of how established or plausible the biological pathway for alcohol use is in its impact on cardiovascular deaths in this region of the world. Britton and McKee [42] were among the first to explain the so-called paradox of alcohol's more detrimental impact in Eastern Europe. The biology is based on substantial amounts of alcohol consumed on irregular heavy drinking occasions, which have been identified as a risk factor for hypertension, ischemic and hemorrhagic stroke, and ischemic heart disease [43, 44], outweighing any beneficial effects of alcohol use on ischemic diseases [45]. This phenomenon can be observed not only in individual-level studies (e.g., [11, 44]) but also in population-level studies [39, 46]. However, most of the evidence was accumulated during periods when more alcohol was consumed in these countries [14], and when extreme heavy drinking occasions were more pronounced (also evidenced by decreases in alcohol poisoning deaths in the region [47, 48]). Large population-based cohort studies in the region with mortality outcomes are needed to ascertain whether these risk relations still prevail. For such studies, it will be important to include people from lower socio-economic strata, and not restrict such studies to the usual cohorts of stable, middle-class professionals with steadier drinking patterns [49, 50].

Such studies could also clarify potential gender differences. One problem with the RR-specific estimates may be that the attributable mortality for women is estimated to be higher than the gold standard, albeit with overlapping CIs (see Table 1).

There are some limitations to this study which need to be enumerated. First and foremost, any modelling is based on assumptions, and the assumptions here may not be completely comparable. While the direct method of estimating PPFs is considered to be the gold standard [51], both the direct and indirect methods of PPF estimation have limitations which may lead to inaccuracies in the respective PPF estimates. The direct method of estimating the effects of decreases in alcohol use on mortality includes causes of death which are not considered to be causally related to alcohol use − however, this method may result in the inclusion of diseases which, in fact, are causally related to alcohol use but where the scientific evidence is not sufficient to determine causality − and will be affected by confounding due to co-occurring changes in other risk factors which are not controlled for [51]. Indirect methods of PPF estimation are based on causes of death that are considered causally related to alcohol use and models use data on alcohol use from population surveys and RRs from case-control and cohort studies. Population surveys are susceptible to numerous biases reflecting both methodological choices and population response patterns, which may affect the estimated prevalence of drinking and the average daily alcohol use statistics by age and sex [52, 53]. The use of RRs does not take into account the interactions of alcohol with the level of country-specific risk factors such as socio-economic status, smoking, body mass index, hepatitis, and diet [50, 54, 55]. Furthermore, RRs from cohort and case-control studies are susceptible to numerous biases due to study design and exposure measurement [56].

The study described herein was conservative in that it excluded all cancer deaths, even though empirical studies from Australia have demonstrated that tobacco and alcohol interventions showed some effects on cancer following implementation, with the full effect accumulating 20 years later [57]. However, most studies observed no effects in the first years after an intervention (e.g., [30, 39]), which is biologically plausible [58]. As for injuries, the impact of alcohol use varies widely (e.g., [59]) and depends much more on social determinants than is the case for chronic diseases, and thus it is recommended that country- or region-specific studies be conducted. However, the findings of the current study also draw attention to the need for the epidemiological profile of alcohol use in a country to be considered for all health outcomes when deciding on which RRs to use to estimate PAFs and PPFs. Other exceptions to the global RRs should be identified and addressed appropriately (e.g., via country-specific RR meta-analyses) and, thus, should be an area of future research. One example is the RR for oesophageal cancer and other cancers impacted by acetaldehyde, which should be modelled based on the distribution of aldehyde dehydrogenase polymorphisms; in countries with a high number of such polymorphisms, such as Japan, the RR is much higher and alcohol-attributable cancer mortality is greater [60].

## Conclusions

The recent 2017 increase in alcohol excise taxes in Lithuania has allowed for a comparison of different indirect methodologies for estimating PPFs with the direct estimates obtained via time-series predictions. The estimates from all indirect methodologies, particularly those based on price elasticities, were markedly lower than those obtained using direct methodologies. With respect to risk relations, the Russia-specific RRs were closer to the direct estimates. Therefore, the current study provided no evidence for a need to change the use of Russia-specific RRs for data from Lithuania. However, more country-specific research is necessary to improve comparative risk analysis methodologies.

## Statement of Ethics

The protocol of this study has been approved by the Centre for Addiction and Mental Health Research Ethics Board (CAMH REB) #050/2020 renewed on March 30, 2021. No human participants; modelling of secondary data. For REB approval, see above.

## Conflict of Interest Statement

The authors have no conflicts of interest to declare.

## Funding Sources

This research has been supported by grants 1R01AA028224 from the US National Institute on Alcohol Abuse and Alcoholism (NIH) and REN 477887 from the Canadian Institutes of Health Research (CIHR INMHA).

## Author Contributions

Jürgen Rehm: conceptualized the study and wrote a first draft, contributed to the modelling and statistical analyses, and helped secure the funding; Huan Jiang, Pol Rovira, Kevin David Shield, Alexander Tran, and Kawon Victoria Kim: contributed to the modelling and statistical analyses; Robin Room, Shannon Lange, and Mindaugas Štelemėkas: helped secure the funding; all authors: contributed to the writing, and approved the final version of the text.

## Data Availability Statement

All calculations are based on official data from Lithuanian agencies named as the respective data sources in the publication. Any further enquiries for the data should be exclusively directed to these agencies. The R programs used to do the statistical calculations can be obtained from the first author.

## Supplementary Material

Supplementary data

## Figures and Tables

**Table 1:** Number of deaths averted and alcohol-attributable fractions following the increase in taxation on March 1,2017 in Lithuania

Deaths averted	Indirect estimate based on APC changes − global RR	Indirect estimate based on APC changes − Russia-specific RR	Indirect estimate based on taxation changes and price elasticity − global RR	Indirect estimate based on taxation changes and price elasticity − Russia-specific RR	Direct estimate based on time-series prediction [20]
Women					
*N* (95% UI)	75 (53–304)	211 (125–313)	41 (35–160)	116 (91–144)	176 (−51 to 404)
PAF (95% UI), %	0.4 (0.3–1.5)	1.0 (0.6–1.5)	0.2 (0.2–0.8)	0.6 (0.4–0.7)	0.9 (−0.2 to 2.0)
Men					
*N* (95% UI)	140 (92–196)	212 (147–295)	65 (53–96)	97 (82–130)	979 (618–1,340)
PAF (95% UI), %	0.7 (0.5–1.0)	1.1 (0.8–1.6)	0.3 (0.3–0.5)	0.5 (0.4–0.7)	5.1 (3.2–7.0)
Total					
*N* (95% UI)	215 (166–444)	423 (322–549)	106 (92–244)	213 (180–261)	1,155 (729–1,582)
PAF (95% UI), %	0.5 (0.4–1.1)	1.1 (0.8–1.4)	0.3 (0.2–0.6)	0.5 (0.5–0.7)	2.9 (1.8–4.0)

APC, adult (15+) alcohol per capita consumption; RR, relative risk; UI, uncertainty interval; PAF, population-attributable fraction for alcohol.

**Table 2:** Deaths averted based on estimated alcohol per capita changes using global relative risks

Deaths averted	Infectious diseases (UI)	Cardiovascular diseases (UI)	GI diseases (UI)	Injuries (UI)	Other diseases (UI)	All diseases (UI)
Women	3 (2–5)	28 (18–254)	13 (7–20)	13 (8–18)	19 (13–25)	77 (53–304)
Men	7 (3–10)	43 (23–89)	14 (9–19)	49 (34–61)	28 (19–38)	140 (92–196)
Total	10 (5–14)	70 (50–295)	26 (18–35)	62 (43–75)	47 (35–59)	215 (166–444)

UI, uncertainty interval; GI, gastrointestinal diseases.

**Table 3:** Deaths averted based on estimated alcohol per capita changes using Russia-specific relative risks

Deaths averted	Infectious diseases (UI)	Cardiovascular diseases (UI)	GI diseases (UI)	Injuries (UI)	Other diseases (UI)	All diseases (UI)
Women	3 (2–5)	170 (93–262)	9 (6–14)	9 (6–14)	19 (14–25)	211 (125–313)
Men	9 (6–13)	131 (82–191)	18 (12–25)	26 (19–35)	28 (19–39)	212 (147–295)
Total	13 (10–17)	301 (213–410)	27 (20–35)	35 (28–45)	47 (37–60)	423 (322–549)

UI, uncertainty interval; GI, gastrointestinal diseases.

## References

[1] Levin ML (1953). The occurrence of lung cancer in man. Acta Unio Int Contra Cancrum.

[2] Rehm J, Kehoe T, Gmel G, Stinson F, Grant B, Gmel G (2010). Statistical modeling of volume of alcohol exposure for epidemiological studies of population health: the US example. Popul Health Metr.

[3] GBD 2019 Risk Factors Collaborators (2020). Global burden of 87 risk factors in 204 countries and territories, 1990–2019: a systematic analysis for the Global Burden of Disease Study 2019. Lancet.

[4] Shield K, Manthey J, Rylett M, Probst C, Wettlaufer A, Parry CDH (2020). National, regional, and global burdens of disease from 2000 to 2016 attributable to alcohol use: a comparative risk assessment study. Lancet Public Health.

[5] Rehm J, Gmel GE, Gmel G, Hasan OSM, Imtiaz S, Popova S (2017). The relationship between different dimensions of alcohol use and the burden of disease-an update. Addiction.

[6] Shield KD, Rehm J (2021). Societal development and the alcohol-attributable burden of disease. Addiction.

[7] Rehm J, Ashley MJ, Room R, Single E, Bondy S, Ferrence R (1996). On the emerging paradigm of drinking patterns and their social and health consequences. Addiction.

[8] Rehm J, Greenfield TK, Kerr W (2006). Patterns of drinking and mortality from different diseases: an overview. Contemp Drug Probl.

[9] World Health Organization (2018). Global status report on alcohol and health 2018.

[10] World Health Organization (2021). Making the European Region Safer: developments in alcohol control policies, 2010–2019. https://www.euro.who.int/en/publications/abstracts/making-the-european-region-safer-developments-in-alcohol-control-policies,-20102019-2021.

[11] Zaridze D, Lewington S, Boroda A, Scelo G, Karpov R, Lazarev A (2014). Alcohol and mortality in Russia: prospective observational study of 151,000 adults. Lancet.

[12] Manthey J, Shield KD, Rylett M, Hasan OSM, Probst C, Rehm J (2019). Global alcohol exposure between 1990 and 2017 and forecasts until 2030: a modelling study. Lancet.

[13] Shield KD, Rehm J (2015). Russia-specific relative risks and their effects on the estimated alcohol-attributable burden of disease. BMC Public Health.

[14] Shield KD, Rylett M, Rehm J (2016). Public health successes and missed opportunities. In: Trends in alcohol consumption and attributable mortality in the WHO European Region, 1990–2014.

[15] The World Bank World Bank analytical classifications for countries. Country analytical history, Country and lending groups. http://www.databank.worldbank.org/data/download/site-content/OGHIST.xls.

[16] OECD (2021). Country Health Profiles 2019. https://www.oecd.org/health/country-health-profiles-eu.htm.

[17] Miščikienė L, Midttun NG, Galkus L, Belian G, Petkevičienė J, Vaitkevičiūtė J (2020). Review of the Lithuanian alcohol control legislation in 1990–2020. Int J Environ Res Public Health.

[18] Stumbrys D, Telksnys T, Jasilionis D, Liutkutė Gumarov V, Galkus L, Goštautaitė Midttun N (2020). Alcohol-related male mortality in the context of changing alcohol control policy in Lithuania 2000–2017. Drug Alcohol Rev.

[19] Štelemėkas M, Manthey J, Badaras R, Casswell S, Ferreira-Borges C, Kalėdienė R (2021). Alcohol control policy measures and all-cause mortality in Lithuania: an interrupted time-series analysis. Addiction.

[20] Tran A, Jiang H, Kim KV, Room R, Štelemėkas M, Lange S (2022). Predicting the impact of alcohol taxation increases on mortality: a comparison of different estimation techniques. Alcohol Alcohol.

[21] Beard E, Marsden J, Brown J, Tombor I, Stapleton J, Michie S (2019). Understanding and using time series analyses in addiction research. Addiction.

[22] Jiang H, Feng X, Lange S, Tran A, Manthey J, Rehm J (2022). Estimating effects of health policy interventions using interrupted time-series analyses: a simulation study. BMC Med Res Methodol.

[23] Jiang H, Tran A, Gmel G, Lange S, Manthey J, Room R (2021). How to quanitfy deaths averted derived from interrupted time-series analyses. https://www.medrxiv.org/content/10.1101/2021.03.23.21254181v1.full.pdf.

[24] Nelson JP, Moran JR (2019). Effects of alcohol taxation on prices: a systematic review and meta-analysis of pass-through rates. The B E J Econ Anal Policy.

[25] Wagenaar AC, Salois MJ, Komro KA (2009). Effects of beverage alcohol price and tax levels on drinking: a meta-analysis of 1,003 estimates from 112 studies. Addiction.

[26] Shield KD, Parkin DM, Whiteman DC, Rehm J, Viallon V, Micallef CM (2016). Population attributable and preventable fractions: cancer risk factor surveillance, and cancer policy projection. Curr Epidemiol Rep.

[27] Ornstein SI, Levy D (1983). Price and income elasticities and the demand for alcoholic beverages. In: American MedicalSociety on alcoholism tRSoA, and the national council on alcoholism. Recent developments in alcoholism: an official publication of the American medical society on alcoholism, the research society on Alcoholism, and the national council on alcoholism 1.

[28] Fogarty J (2010). The demand for beer, wine and spirits: a survey of the literature. J Econ Surv.

[29] Rehm J, Patra J, Popova S (2007). Alcohol drinking cessation and its effect on esophageal and head and neck cancers: a pooled analysis. Int J Cancer.

[30] Grundy A, Poirier AE, Khandwala F, Grevers X, Friedenreich CM, Brenner DR (2017). Cancer incidence attributable to lifestyle and environmental factors in Alberta in 2012: summary of results. CMAJ Open.

[31] Jiang H, Livingston M, Room R, Chenhall R, English DR (2018). Temporal associations of alcohol and tobacco consumption with cancer mortality. JAMA Netw Open.

[32] Rumgay H, Shield K, Charvat H, Ferrari P, Sornpaisarn B, Obot I (2021). Global burden of cancer in 2020 attributable to alcohol consumption: a population-based study. Lancet Oncol.

[33] Churchill S, Angus C, Purshouse R, Brennan A, Sherk A (2020). Expanding attributable fraction applications to outcomes wholly attributable to a risk factor. Stat Methods Med Res.

[34] Powell M, GSaH JP (1994). A direct search optimization method that models the objective and constraint functions by linear interpolation. Advances in optimization and numerical analysis.

[35] Lithuanian Department of Statistics (2021). Official Data Portal. Legal alcohol consumption per person aged 15 and older. https://osp.stat.gov.lt/statistiniu-rodikliu-analize#/.

[36] Graham C, Talay D (2013). Stochastic simulation and Monte Carlo methods: mathematical foundations of stochastic simulation.

[37] Gmel G, Shield KD, Frick H, Kehoe T, Gmel G, Rehm J (2011). Estimating uncertainty of alcohol-attributable fractions for infectious and chronic diseases. BMC Med Res Methodol.

[38] R Core Team. (2014). R. A language and environment for statistical computing. Version 4.0.1.

[39] Leon DA, Chenet L, Shkolnikov VM, Zakharov S, Shapiro J, Rakhmanova G (1997). Huge variation in Russian mortality rates 1984–1994: artefact, alcohol, or what?. Lancet.

[40] Hamilton JD (2020). Time series analysis.

[41] Rehm J, Tran A, Gobiņa I, Janik-Koncewicz K, Jiang H, Kim KV (2022). Do alcohol control policies have the predicted effects on consumption? An analysis of the Baltic countries and Poland 2000–2020. Drug Alcohol Depend.

[42] Britton A, McKee M (2000). The relation between alcohol and cardiovascular disease in Eastern Europe: explaining the paradox. J Epidemiol Community Health.

[43] Rehm J, Roerecke M (2017). Cardiovascular effects of alcohol consumption. Trends Cardiovasc Med.

[44] Leon DA, Shkolnikov VM, McKee M, Kiryanov N, Andreev E (2010). Alcohol increases circulatory disease mortality in Russia: acute and chronic effects or misattribution of cause?. Int J Epidemiol.

[45] Roerecke M, Rehm J (2014). Alcohol consumption, drinking patterns, and ischemic heart disease: a narrative review of meta-analyses and a systematic review and meta-analysis of the impact of heavy drinking occasions on risk for moderate drinkers. BMC Med.

[46] Rehm J, Room R, Monteiro M, Gmel G, Graham K, Rehn N, Ezzati ADL M, Rodgers A, Murray CJL (2004). Alcohol use. Comparative quantification of health risks global and regional burden of disease attributable to selected major risk factors.

[47] Global Health Data Exchange (2020). GBD 2019 results tool (online).

[48] Nemtsov A, Neufeld M, Rehm J (2019). Are trends in alcohol consumption and cause-specific mortality in Russia between 1990 and 2017 the result of alcohol policy measures?. J Stud Alcohol Drugs.

[49] Rehm J (2019). Why the relationship between level of alcohol-use and all-cause mortality cannot be addressed with meta-analyses of cohort studies. Drug Alcohol Rev.

[50] Probst C, Kilian C, Sanchez S, Lange S, Rehm J (2020). The role of alcohol use and drinking patterns in socioeconomic inequalities in mortality: a systematic review. Lancet Public Health.

[51] Norström T, Skog OJ (2001). Alcohol and mortality: methodological and analytical issues in aggregate analyses. Addiction.

[52] Groves RM (2004). Survey errors and survey costs. (Wiley series in survey methodology).

[53] Shield KD, Rehm J (2012). Difficulties with telephone-based surveys on alcohol consumption in high-income countries: the Canadian example. Int J Methods Psychiatr Res.

[54] Beynon RA, Lang S, Schimansky S, Penfold CM, Waylen A, Thomas SJ (2018). Tobacco smoking and alcohol drinking at diagnosis of head and neck cancer and all-cause mortality: results from head and neck 5,000, a prospective observational cohort of people with head and neck cancer. Int J Cancer.

[55] Loomba R, Yang H-I, Su J, Brenner D, Barrett-Connor E, Iloeje U (2013). Synergism between obesity and alcohol in increasing the risk of hepatocellular carcinoma: a prospective cohort study. Am J Epidemiol.

[56] Naimi TS, Stockwell T, Zhao J, Xuan Z, Dangardt F, Saitz R (2017). Selection biases in observational studies affect associations between “moderate”alcohol consumption and mortality. Addiction.

[57] Jiang H, Livingston M, Room R, Gan Y, English D, Chenhall R (2019). Can public health policies on alcohol and tobacco reduce a cancer epidemic? Australia's experience. BMC Med.

[58] IARC Working Group on the Evaluation of Carcinogenic Risks to Humans (2012). Personal habits and indoor combustions. IARC Monogr Eval Carcinog Risks Hum.

[59] Cherpitel CJ, Ye Y, Bond J, Rehm J, Poznyak V, Macdonald S (2005). Multi-level analysis of alcohol-related injury among emergency department patients: a cross-national study. Addiction.

[60] Roerecke M, Shield KD, Higuchi S, Yoshimura A, Larsen E, Rehm MX (2015). Estimates of alcohol-related oesophageal cancer burden in Japan: systematic review and meta-analyses. Bull World Health Organ.

