# Supplementary material for: Using Direct and Indirect Estimates for Alcohol-Attributable Mortality: A Modelling Study Using the Example of Lithuania
Source: Eur Addict Res. 2023 Feb 7;29(2):119–26. doi: 10.1159/000529200 (PMC10238643; doi:10.1159/000529200)
Supplement: Supplementary file 1 — Supplementary data [file ear-0029-0119-s01.docx]

# Supplementary materials:

# Estimating direct and indirect alcohol-attributable mortality: a modelling study using the example of Lithuania

## Table S1: Alcohol-attributable categories and ICD codes

| **Disease** | **Sex (Age)** | **Global RR** | **Russian RR** |
| --- | --- | --- | --- |
| Tuberculosis | Men | ${RR}_{CD}=exp(\beta_{1}x$)  β_1_ = 0.0179695 | If(x < 25.36):  ${RR}_{CD}=1.01$  If(25.36 ≤ x < 76.08):  ${RR}_{CD}=1.97$  If(76.08 ≤ x):  ${RR}_{CD}=4.14$ |
| Tuberculosis | Women | ${RR}_{CD}=exp(\beta_{1}x$)  β_1_ = 0.0179695 | If(x < 25.36):  ${RR}_{CD}=1.01$  If(25.36 ≤ x < 76.08):  ${RR}_{CD}=4.06$  If(76.08 ≤ x):  ${RR}_{CD}=5.32$ |
| HIV/AIDS | Men | If(x ≤ 61):  ${RR}_{CD}=1$  If(x > 61):  ${RR}_{CD}=1.54$ | If(x ≤ 61):  ${RR}_{CD}=1$  If(x > 61):  ${RR}_{CD}=1.54$ |
| HIV/AIDS | Women | If(x ≤ 49):  ${RR}_{CD}=1$  If(x > 49):  ${RR}_{CD}=1.54$ | If(x ≤ 49):  ${RR}_{CD}=1$  If(x > 49):  ${RR}_{CD}=1.54$ |
| Lower respiratory infections | Men | ${RR}_{CD}=exp(\beta_{1}(\frac{x+y_{1}}{100})$)  β_1_ = 0.4764038  y_1_ = 0.0399999618530273 | If(x < 25.36):  ${RR}_{CD}=0.95$  If(25.36 ≤ x < 76.08):  ${RR}_{CD}=1.92$  If(76.08 ≤ x):  ${RR}_{CD}=3.29$ |
| Lower respiratory infections | Women | ${RR}_{CD}=exp(\beta_{1}(\frac{x+y_{1}}{100})$)  β_1_ = 0.4764038  y_1_ = 0.0399999618530273 | If(x < 25.36):  ${RR}_{CD}=2.10$  If(25.36 ≤ x < 76.08):  ${RR}_{CD}=3.21$  If(76.08 ≤ x):  ${RR}_{CD}=3.42$ |
| Hypertensive heart disease | Men | If(x ≤ 21):  ${RR}_{CD}=exp(\beta_{1}\cdot x+\beta_{2}\cdot\frac{x^{3}}{{75}^{2}})$  If(21 ≤ x < 75):  ${RR}_{CD}=exp(\beta_{1}x+\beta_{2}\frac{x^{3}-\frac{{75(x-21)}^{3}}{(75-21)}}{{75}^{2}})$  If(75 ≤ x):  ${RR}_{CD}=exp(\beta_{1}x+\beta_{2}\frac{x^{3}-\frac{{75(x-21)}^{3}-{21(x-75)}^{3}}{(75-21)}}{{75}^{2}})$  β_1_ = 0.0150537  β_1_ = -0.0156155 | If(x ≤ 21):  ${RR}_{CD}=exp(\beta_{1}\cdot x+\beta_{2}\cdot\frac{x^{3}}{{75}^{2}})$  If(21 ≤ x < 75):  ${RR}_{CD}=exp(\beta_{1}x+\beta_{2}\frac{x^{3}-\frac{{75(x-21)}^{3}}{(75-21)}}{{75}^{2}})$  If(75 ≤ x):  ${RR}_{CD}=exp(\beta_{1}x+\beta_{2}\frac{x^{3}-\frac{{75(x-21)}^{3}-{21(x-75)}^{3}}{(75-21)}}{{75}^{2}})$  β_1_ = 0.0150537  β_1_ = -0.0156155 |
| Hypertensive heart disease | Women | If(x ≤ 18.9517):  ${RR}_{CD}=1$  If(18.9517 ≤ x < 75):  ${RR}_{CD}=exp(\beta_{1}x+\beta_{2}\frac{x^{3}-\frac{{20(x-10)}^{3}-{10(x-20)}^{3}}{(20-10)}}{{20}^{2}})$  If(75 ≤ x):  ${RR}_{CD}=exp({75\beta}_{1}+\beta_{2}\frac{{75}^{3}-\frac{{20(75-10)}^{3}-{10(75-20)}^{3}}{(20-10)}}{{20}^{2}})$  β_1_ = -0.0154196  β_1_ = 0.0217586 | If(x ≤ 18.9517):  ${RR}_{CD}=1$  If(18.9517 ≤ x < 75):  ${RR}_{CD}=exp(\beta_{1}x+\beta_{2}\frac{x^{3}-\frac{{20(x-10)}^{3}-{10(x-20)}^{3}}{(20-10)}}{{20}^{2}})$  If(75 ≤ x):  ${RR}_{CD}=exp({75\beta}_{1}+\beta_{2}\frac{{75}^{3}-\frac{{20(75-10)}^{3}-{10(75-20)}^{3}}{(20-10)}}{{20}^{2}})$  β_1_ = -0.0154196  β_1_ = 0.0217586 |
| Ischaemic heart disease | Men  (15-34 years old) | If(x < 60):  ${RR}_{CD}=\exp\left( \beta_{1}\left( \beta_{2}\sqrt{y_{1}}+\beta_{3}{y_{1}}^{3} \right) \right)$  If(60 ≤ x < 100):  ${RR}_{CD}=y_{2}+exp\left( \beta_{1}\left( \beta_{2}\sqrt{y_{3}}+\beta_{3}{y_{3}}^{3} \right) \right)$  If(100 ≤ x):  ${RR}_{CD}=\exp\left( \beta_{4}\left( x-100 \right) \right)-1+y_{2}+exp\left( \beta_{1}\left( \beta_{2}\sqrt{y_{3}}+\beta_{3}{y_{3}}^{3} \right) \right)$  β_1_ = 1.111874  β_2_ = -0.4870068  β_3_ = 1.550984  β_4_ = 0.012  $y_{1}=\frac{x+0.0099999997764826}{100}$  $y_{2}=0.04571551$  $y_{3}=\frac{60+0.0099999997764826}{100}$ | If(x < 25.36):  ${RR}_{CD}=1.09178$  If(25.36 ≤ x < 76.08):  ${RR}_{CD}=1.49618$  If(76.08 ≤ x):  ${RR}_{CD}=2.43944$ |
| Ischaemic heart disease | Men  (35-64 years old) | If(x < 60):  ${RR}_{CD}=\exp\left( \beta_{1}\left( \beta_{2}\sqrt{y_{1}}+\beta_{3}{y_{1}}^{3} \right) \right)$  If(60 ≤ x < 100):  ${RR}_{CD}=y_{2}+exp\left( \beta_{1}\left( \beta_{2}\sqrt{y_{3}}+\beta_{3}{y_{3}}^{3} \right) \right)$  If(100 ≤ x):  ${RR}_{CD}=\exp\left( \beta_{4}\left( x-100 \right) \right)-1+y_{2}+exp\left( \beta_{1}\left( \beta_{2}\sqrt{y_{3}}+\beta_{3}{y_{3}}^{3} \right) \right)$  β_1_ = 0.757104  β_2_ = -0.4870068  β_3_ = 1.550984  β_4_ = 0.012  $y_{1}=\frac{x+0.0099999997764826}{100}$  $y_{2}=0.04571551$  $y_{3}=\frac{60+0.0099999997764826}{100}$ | If(x < 25.36):  ${RR}_{CD}=1.09178$  If(25.36 ≤ x < 76.08):  ${RR}_{CD}=1.49618$  If(76.08 ≤ x):  ${RR}_{CD}=2.43944$ |
| Ischaemic heart disease | Men  (+65 years old) | If(x < 60):  ${RR}_{CD}=\exp\left( \beta_{1}\left( \beta_{2}\sqrt{y_{1}}+\beta_{3}{y_{1}}^{3} \right) \right)$  If(60 ≤ x < 100):  ${RR}_{CD}=y_{2}+exp\left( \beta_{1}\left( \beta_{2}\sqrt{y_{3}}+\beta_{3}{y_{3}}^{3} \right) \right)$  If(100 ≤ x):  ${RR}_{CD}=\exp\left( \beta_{4}\left( x-100 \right) \right)-1+y_{2}+exp\left( \beta_{1}\left( \beta_{2}\sqrt{y_{3}}+\beta_{3}{y_{3}}^{3} \right) \right)$  β_1_ = 1.035623  β_2_ = -0.4870068  β_3_ = 1.550984  β_4_ = 0.012  $y_{1}=\frac{x+0.0099999997764826}{100}$  $y_{2}=0.04571551$  $y_{3}=\frac{60+0.0099999997764826}{100}$ | If(x < 25.36):  ${RR}_{CD}=1.09178$  If(25.36 ≤ x < 76.08):  ${RR}_{CD}=1.49618$  If(76.08 ≤ x):  ${RR}_{CD}=2.43944$ |
| Ischaemic heart disease | Women  (15-34 years old) | If(x < 30.3814):  ${RR}_{CD}=\exp\left( \beta_{1}\left( \beta_{2}y_{1}+\beta_{3}y_{1}\ln(y_{1}) \right) \right)$  If(30.3814 ≤ x):  ${RR}_{CD}=\exp\left( \beta_{4}\left( x-30.3814 \right) \right)-1+\exp\left( \beta_{1}\left( \beta_{2}y_{2}+\beta_{3}y_{2}\ln(y_{2}) \right) \right)$  β_1_ = 1.111874  β_2_ = 1.832441  β_3_ = 1.538557  β_4_ = 0.01  $y_{1}=\frac{x+0.0099999997764826}{100}$  $y_{2}=\frac{30.3814+0.0099999997764826}{100}$ | If(x < 25.36):  ${RR}_{CD}=1.51383$  If(25.36 ≤ x < 76.08):  ${RR}_{CD}=3.43525$  If(76.08 ≤ x):  ${RR}_{CD}=7.41902$ |
| Ischaemic heart disease | Women  (35-64 years old) | If(x < 30.3814):  ${RR}_{CD}=\exp\left( \beta_{1}\left( \beta_{2}y_{1}+\beta_{3}y_{1}\ln(y_{1}) \right) \right)$  If(30.3814 ≤ x):  ${RR}_{CD}=\exp\left( \beta_{4}\left( x-30.3814 \right) \right)-1+\exp\left( \beta_{1}\left( \beta_{2}y_{2}+\beta_{3}y_{2}\ln(y_{2}) \right) \right)$  β_1_ = 1.035623  β_2_ = 1.832441  β_3_ = 1.538557  β_4_ = 0.009300  $y_{1}=\frac{x+0.0099999997764826}{100}$  $y_{2}=\frac{30.3814+0.0099999997764826}{100}$ | If(x < 25.36):  ${RR}_{CD}=1.51383$  If(25.36 ≤ x < 76.08):  ${RR}_{CD}=3.43525$  If(76.08 ≤ x):  ${RR}_{CD}=7.41902$ |
| Ischaemic heart disease | Women  (+65 years old) | If(x < 30.3814):  ${RR}_{CD}=\exp\left( \beta_{1}\left( \beta_{2}y_{1}+\beta_{3}y_{1}\ln(y_{1}) \right) \right)$  If(30.3814 ≤ x):  ${RR}_{CD}=\exp\left( \beta_{4}\left( x-30.3814 \right) \right)-1+\exp\left( \beta_{1}\left( \beta_{2}y_{2}+\beta_{3}y_{2}\ln(y_{2}) \right) \right)$  β_1_ = 0.757104  β_2_ = 1.832441  β_3_ = 1.538557  β_4_ = 0.0068  $y_{1}=\frac{x+0.0099999997764826}{100}$  $y_{2}=\frac{30.3814+0.0099999997764826}{100}$ | If(x < 25.36):  ${RR}_{CD}=1.51383$  If(25.36 ≤ x < 76.08):  ${RR}_{CD}=3.43525$  If(76.08 ≤ x):  ${RR}_{CD}=7.41902$ |
| Ischemic stroke | Men  (15-34 years old) | If(x ≤ 1):  ${RR}_{CD}=1-x(1-\exp\left( \beta_{1}\left( \beta_{2}\sqrt{y_{1}}+\beta_{3}\sqrt{y_{1}}\ln\left( y_{1} \right) \right) \right))$  If(1 < x):  ${RR}_{CD}=\exp\left( \beta_{1}\left( \beta_{2}\sqrt{y_{2}}+\beta_{3}\sqrt{y_{2}}\ln\left( y_{2} \right) \right) \right)$  β_1_ = 1.111874  β_2_ = 0.4030081  β_3_ = 0.3877538  $y_{1}=\frac{1+0.0028572082519531}{100}$  $y_{2}=\frac{x+0.0028572082519531}{100}$ | If(x < 25.36):  ${RR}_{CD}=1.06$  If(25.36 ≤ x < 76.08):  ${RR}_{CD}=1.14$  If(76.08 ≤ x):  ${RR}_{CD}=1.28$ |
| Ischemic stroke | Men  (35-64 years old) | If(x ≤ 1):  ${RR}_{CD}=1-x(1-\exp\left( \beta_{1}\left( \beta_{2}\sqrt{y_{1}}+\beta_{3}\sqrt{y_{1}}\ln\left( y_{1} \right) \right) \right))$  If(1 < x):  ${RR}_{CD}=\exp\left( \beta_{1}\left( \beta_{2}\sqrt{y_{2}}+\beta_{3}\sqrt{y_{2}}\ln\left( y_{2} \right) \right) \right)$  β_1_ = 1.035623  β_2_ = 0.4030081  β_3_ = 0.3877538  $y_{1}=\frac{1+0.0028572082519531}{100}$  $y_{2}=\frac{x+0.0028572082519531}{100}$ | If(x < 25.36):  ${RR}_{CD}=1.06$  If(25.36 ≤ x < 76.08):  ${RR}_{CD}=1.14$  If(76.08 ≤ x):  ${RR}_{CD}=1.28$ |
| Ischemic stroke | Men  (+65 years old) | If(x ≤ 1):  ${RR}_{CD}=1-x(1-\exp\left( \beta_{1}\left( \beta_{2}\sqrt{y_{1}}+\beta_{3}\sqrt{y_{1}}\ln\left( y_{1} \right) \right) \right))$  If(1 < x):  ${RR}_{CD}=\exp\left( \beta_{1}\left( \beta_{2}\sqrt{y_{2}}+\beta_{3}\sqrt{y_{2}}\ln\left( y_{2} \right) \right) \right)$  β_1_ = 0.757104  β_2_ = 0.4030081  β_3_ = 0.3877538  $y_{1}=\frac{1+0.0028572082519531}{100}$  $y_{2}=\frac{x+0.0028572082519531}{100}$ | If(x < 25.36):  ${RR}_{CD}=1.06$  If(25.36 ≤ x < 76.08):  ${RR}_{CD}=1.14$  If(76.08 ≤ x):  ${RR}_{CD}=1.28$ |
| Ischemic stroke | Women  (15-34 years old) | If(x ≤ 1):  ${RR}_{CD}=1-x(1-\exp\left( \beta_{1}\left( \beta_{2}\sqrt{y_{1}}+\beta_{3}y_{1} \right) \right))$  If(1 < x):  ${RR}_{CD}=\exp\left( \beta_{1}\left( \beta_{2}\sqrt{y_{2}}+\beta_{3}y_{2} \right) \right)$  β_1_ = 1.111874  β_2_ = -2.48768  β_3_ = 3.7087240  $y_{1}=\frac{1+0.0028572082519531}{100}$  $y_{2}=\frac{x+0.0028572082519531}{100}$ | If(x < 25.36):  ${RR}_{CD}=1.38$  If(25.36 ≤ x < 76.08):  ${RR}_{CD}=1.36$  If(76.08 ≤ x):  ${RR}_{CD}=1.62$ |
| Ischemic stroke | Women  (35-64 years old) | If(x ≤ 1):  ${RR}_{CD}=1-x(1-\exp\left( \beta_{1}\left( \beta_{2}\sqrt{y_{1}}+\beta_{3}y_{1} \right) \right))$  If(1 < x):  ${RR}_{CD}=\exp\left( \beta_{1}\left( \beta_{2}\sqrt{y_{2}}+\beta_{3}y_{2} \right) \right)$  β_1_ = 1.035623  β_2_ = -2.48768  β_3_ = 3.7087240  $y_{1}=\frac{1+0.0028572082519531}{100}$  $y_{2}=\frac{x+0.0028572082519531}{100}$ | If(x < 25.36):  ${RR}_{CD}=1.38$  If(25.36 ≤ x < 76.08):  ${RR}_{CD}=1.36$  If(76.08 ≤ x):  ${RR}_{CD}=1.62$ |
| Ischemic stroke | Women  (+65 years old) | If(x ≤ 1):  ${RR}_{CD}=1-x(1-\exp\left( \beta_{1}\left( \beta_{2}\sqrt{y_{1}}+\beta_{3}y_{1} \right) \right))$  If(1 < x):  ${RR}_{CD}=\exp\left( \beta_{1}\left( \beta_{2}\sqrt{y_{2}}+\beta_{3}y_{2} \right) \right)$  β_1_ = 0.757104  β_2_ = -2.48768  β_3_ = 3.7087240  $y_{1}=\frac{1+0.0028572082519531}{100}$  $y_{2}=\frac{x+0.0028572082519531}{100}$ | If(x < 25.36):  ${RR}_{CD}=1.38$  If(25.36 ≤ x < 76.08):  ${RR}_{CD}=1.36$  If(76.08 ≤ x):  ${RR}_{CD}=1.62$ |
| Intracerebral hemorrhage,  Subarachnoid hemorrhage | Men | If(x ≤ 1):  ${RR}_{CD}=1-x(1-exp\left( \beta_{1}\left( \frac{1+y_{1}}{100} \right) \right))$  If(1 < x):  ${RR}_{CD}=\exp\left( \beta_{1}\left( \frac{1+y_{1}}{100} \right) \right)$  β_1_ = 0.6898937  y_1_ = 0.0028572082519531 | If(x < 25.36):  ${RR}_{CD}=1.06$  If(25.36 ≤ x < 76.08):  ${RR}_{CD}=1.14$  If(76.08 ≤ x):  ${RR}_{CD}=1.28$ |
| Intracerebral hemorrhage,  Subarachnoid hemorrhage | Women | If(x ≤ 1):  ${RR}_{CD}=1-x(1-exp\left( \beta_{1}\left( \frac{1+y_{1}}{100} \right) \right))$  If(1 < x):  ${RR}_{CD}=\exp\left( \beta_{1}\left( \frac{1+y_{1}}{100} \right) \right)$  β_1_ = 1.466406  y_1_ = 0.0028572082519531 | If(x < 25.36):  ${RR}_{CD}=1.38$  If(25.36 ≤ x < 76.08):  ${RR}_{CD}=1.36$  If(76.08 ≤ x):  ${RR}_{CD}=1.62$ |
| Atrial fibrillation and flutter | Both | ${RR}_{CD}=exp(\beta_{1}(\frac{x+y_{1}}{10})$)  β_1_ = 0.0575183  y_1_ = 0.0499992370605469 | ${RR}_{CD}=exp(\beta_{1}(\frac{x+y_{1}}{10})$)  β_1_ = 0.0575183  y_1_ = 0.0499992370605469 |
| Alcoholic cardiomyopathy | Both | 100% Alcohol Attributable | 100% Alcohol Attributable |
| Pancreatitis | Men | ${RR}_{CD}=exp(\beta_{1}x$)  β_1_ = 0.0173451 | If(x < 25.36):  ${RR}_{CD}=1.43$  If(25.36 ≤ x < 76.08):  ${RR}_{CD}=2.07$  If(76.08 ≤ x):  ${RR}_{CD}=6.69$ |
| Pancreatitis | Women | If(x < 3):  ${RR}_{CD}=exp(\beta_{1}\cdot x)$  If(3 ≤ x < 15):  ${RR}_{CD}=exp(\beta_{1}x+\beta_{2}\frac{{(x-3)}^{3}}{{(40-3)}^{2}})$  If(15 ≤ x < 40):  ${RR}_{CD}=exp(\beta_{1}x+\beta_{2}\frac{{(x-3)}^{3}-\frac{\left( x-15 \right)^{3}(40-3)}{(40-15)}}{{(40-3)}^{2}})$  If(40 ≤ x < 108):  ${RR}_{CD}=exp(\beta_{1}x+\beta_{2}\frac{{(x-3)}^{3}-\frac{\left( x-15 \right)^{3}\left( 40-3 \right)}{\left( 40-15 \right)}-\frac{\left( x-40 \right)^{3}(15-3)}{(40-15)}}{{(40-3)}^{2}})$  If(108 ≤ x):  ${RR}_{CD}=exp({108\beta}_{1}+\beta_{2}\frac{{(108-3)}^{3}-\frac{\left( 108-15 \right)^{3}\left( 40-3 \right)}{\left( 40-15 \right)}-\frac{\left( 108-40 \right)^{3}(15-3)}{(40-15)}}{{(40-3)}^{2}})$  β_1_ = -0.0272886  β_1_ = 0.0611466 | If(x < 25.36):  ${RR}_{CD}=1.09$  If(25.36 ≤ x < 76.08):  ${RR}_{CD}=5.01$  If(76.08 ≤ x):  ${RR}_{CD}=19.26$ |
| Liver cirrhosis | Men | If(x ≤ 1):  ${RR}_{CD}=1+x(exp\left( \left( \beta_{1}+\beta_{2} \right)\left( \frac{1+y_{1}}{100} \right) \right)-1)$  If(1 < x):  ${RR}_{CD}=\exp\left( \left( \beta_{1}+\beta_{2} \right)\left( \frac{1+y_{1}}{100} \right) \right)$  β_1_ = 1.687111  β_2_ = 1.106413  y_1_ = 0.1699981689453125 | If(x < 25.36):  ${RR}_{CD}=0.92$  If(25.36 ≤ x < 76.08):  ${RR}_{CD}=1.77$  If(76.08 ≤ x):  ${RR}_{CD}=6.21$ |
| Liver cirrhosis | Women | If(x ≤ 1):  ${RR}_{CD}=1+x(exp\left( \left( \beta_{1}+\beta_{2} \right)\sqrt{\left( \frac{1+y_{1}}{100} \right)} \right)-1)$  If(1 < x):  ${RR}_{CD}=\exp\left( \left( \beta_{1}+\beta_{2} \right)\sqrt{\left( \frac{1+y_{1}}{100} \right)} \right)$  β_1_ = 2.351821  β_2_ = 0.9002139  y_1_ = 0.1699981689453125 | If(x < 25.36):  ${RR}_{CD}=2.50$  If(25.36 ≤ x < 76.08):  ${RR}_{CD}=7.07$  If(76.08 ≤ x):  ${RR}_{CD}=12.08$ |
| Road injuries | Men | Non-heavy episodic drinkers:  ${RR}_{CD}=exp(\beta_{1}x$)  β_1_ = 0.00299550897979837  Heavy episodic drinkers:  ${RR}_{CD}=exp(\beta_{1}x+\beta_{2}$)  β_1_ = 0.00299550897979837  β_2_ = 0.959350221334602 | If(x < 25.36):  ${RR}_{CD}=1.52$  If(25.36 ≤ x < 76.08):  ${RR}_{CD}=2.68$  If(76.08 ≤ x):  ${RR}_{CD}=4.20$ |
| Road injuries | Women | Non-heavy episodic drinkers:  ${RR}_{CD}=exp(\beta_{1}x$)  β_1_ = 0.00299550897979837  Heavy episodic drinkers:  ${RR}_{CD}=exp(\beta_{1}x+\beta_{2}$)  β_1_ = 0.00299550897979837  β_2_ = 0.959350221334602 | If(x < 25.36):  ${RR}_{CD}=1.98$  If(25.36 ≤ x < 76.08):  ${RR}_{CD}=4.48$  If(76.08 ≤ x):  ${RR}_{CD}=3.17$ |
| Poisonings, Falls, Fire, heat, and hot substances, Drowning, Exposure to mechanical forces, Other unintentional injuries | Men | Non-heavy episodic drinkers:  ${RR}_{CD}=exp(\beta_{1}x$)  β_1_ = 0.00199800266267306  Heavy episodic drinkers:  ${RR}_{CD}=exp(\beta_{1}x+\beta_{2}$)  β_1_ = 0.00199800266267306  β_2_ = 0.647103242058538 | If(x < 25.36):  ${RR}_{CD}=1.58$  If(25.36 ≤ x < 76.08):  ${RR}_{CD}=2.48$  If(76.08 ≤ x):  ${RR}_{CD}=6.07$ |
| Poisonings, Falls, Fire, heat, and hot substances, Drowning, Exposure to mechanical forces, Other unintentional injuries | Women | Non-heavy episodic drinkers:  ${RR}_{CD}=exp(\beta_{1}x$)  β_1_ = 0.00199800266267306  Heavy episodic drinkers:  ${RR}_{CD}=exp(\beta_{1}x+\beta_{2}$)  β_1_ = 0.00199800266267306  β_2_ = 0.647103242058538 | If(x < 25.36):  ${RR}_{CD}=2.08$  If(25.36 ≤ x < 76.08):  ${RR}_{CD}=5.24$  If(76.08 ≤ x):  ${RR}_{CD}=8.56$ |
| Self-harm | Men | Non-heavy episodic drinkers:  ${RR}_{CD}=exp(\beta_{1}x$)  β_1_ = 0.00199800266267306  Heavy episodic drinkers:  ${RR}_{CD}=exp(\beta_{1}x+\beta_{2}$)  β_1_ = 0.00199800266267306  β_2_ = 0.647103242058538 | If(x < 25.36):  ${RR}_{CD}=1.21$  If(25.36 ≤ x < 76.08):  ${RR}_{CD}=3.47$  If(76.08 ≤ x):  ${RR}_{CD}=8.62$ |
| Self-harm | Women | Non-heavy episodic drinkers:  ${RR}_{CD}=exp(\beta_{1}x$)  β_1_ = 0.00199800266267306  Heavy episodic drinkers:  ${RR}_{CD}=exp(\beta_{1}x+\beta_{2}$)  β_1_ = 0.00199800266267306  β_2_ = 0.647103242058538 | If(x < 25.36):  ${RR}_{CD}=2.82$  If(25.36 ≤ x < 76.08):  ${RR}_{CD}=8.22$  If(76.08 ≤ x):  ${RR}_{CD}=14.57$ |
| Interpersonal injuries | Men | Non-heavy episodic drinkers:  ${RR}_{CD}=exp(\beta_{1}x$)  β_1_ = 0.00199800266267306  Heavy episodic drinkers:  ${RR}_{CD}=exp(\beta_{1}x+\beta_{2}$)  β_1_ = 0.00199800266267306  β_2_ = 0.647103242058538 | If(x < 25.36):  ${RR}_{CD}=1.75$  If(25.36 ≤ x < 76.08):  ${RR}_{CD}=3.67$  If(76.08 ≤ x):  ${RR}_{CD}=9.47$ |
| Interpersonal injuries | Women | Non-heavy episodic drinkers:  ${RR}_{CD}=exp(\beta_{1}x$)  β_1_ = 0.00199800266267306  Heavy episodic drinkers:  ${RR}_{CD}=exp(\beta_{1}x+\beta_{2}$)  β_1_ = 0.00199800266267306  β_2_ = 0.647103242058538 | If(x < 25.36):  ${RR}_{CD}=3.55$  If(25.36 ≤ x < 76.08):  ${RR}_{CD}=10.23$  If(76.08 ≤ x):  ${RR}_{CD}=19.11$ |
| Alcohol use disorders | Both | 100% Alcohol Attributable | 100% Alcohol Attributable |
| Epilepsy | Both | ${RR}_{CD}=exp(\beta_{1}(\frac{x+0.5}{100})$)  β_1_ = 1.22861 | ${RR}_{CD}=exp(\beta_{1}(\frac{x+0.5}{100})$)  β_1_ = 1.22861 |
| Diabetes | Men | ${RR}_{CD}=exp(\beta_{1}\left( \frac{x}{100} \right)^{2}+\beta_{2}\left( \frac{x}{100} \right)^{3}$)  β_1_ = 0.1763703  β_1_ = -0.0728256 | ${RR}_{CD}=exp(\beta_{1}\left( \frac{x}{100} \right)^{2}+\beta_{2}\left( \frac{x}{100} \right)^{3}$)  β_1_ = 0.1763703  β_1_ = -0.0728256 |
| Diabetes | Women | ${RR}_{CD}=exp(\beta_{1}\sqrt{\left( \frac{x}{100} \right)}+\beta_{2}\left( \frac{x}{100} \right)$)  β_1_ = -1.3133910  β_1_ = 1.0142390 | ${RR}_{CD}=exp(\beta_{1}\sqrt{\left( \frac{x}{100} \right)}+\beta_{2}\left( \frac{x}{100} \right)$)  β_1_ = -1.3133910  β_1_ = 1.0142390 |

For more details, see (5); for risk relations for all categories which are not 100% alcohol attributable, see (4).

- Infectious diseases:
  - Tuberculosis (ICD-10 coding: A10-A14, A15-A18.89, A19-A19.9)
  - HIV/AIDS (ICD-10 coding: B20-B23.8, B24-B24.0)
  - Lower respiratory infections (ICD-10 coding: J09-J18.2, J18.8-J18.9, J19.6-J22.9, J85.1, J91.0, P23-P23.9, U04-U04.9)
- Cardiovascular diseases:
  - Hypertensive heart disease (ICD-10 coding: I11-I11.2, I11.9)
  - Ischaemic heart disease (ICD-10 coding: I20-I21.6, I21.9-I25.9)
  - Ischemic stroke (ICD-10 coding: G45-G46.8, I63-I63.9, I65-I66.9, I67.2-I67.848, I69.3-I69.4)
  - Intracerebral hemorrhage (ICD-10 coding: I61-I62, I62.9, I69.0-I69.298)
  - Subarachnoid hemorrhage (ICD-10 coding: I60-I60.9, I67.0-I67.1)
  - Atrial fibrillation and flutter (ICD-10 coding: I48-I48.92)
  - Alcoholic cardiomyopathy (ICD-10 coding: I42.6)
- Gastrointestinal diseases:
  - Pancreatitis (ICD-10 coding: K85-K86.9)
  - Liver cirrhosis (ICD-10 coding: K70-K71, K71.3-K72, K72.1-K75, K75.2, K75.4-K76.2, K76.4-K77.8)
- Injuries:
  - Road injuries (ICD-10 coding: V01-V04.99, V06-V80.929, V82-V82.9, V87.2-V87.3)
  - Poisonings (ICD-10 coding: X40-X44.9, X46-X49.9)
  - Falls (ICD-10 coding: W00-W19.9)
  - Fire, heat, and hot substances (ICD-10 coding: X00-X06.9, X08-X19.9)
  - Drowning (ICD-10 coding: W65-W70.9, W73-W74.9)
  - Exposure to mechanical forces (ICD-10 coding: W20-W38.9, W40-W43.9, W45.0-W45.2, W46-W46.2, W49-W52)
  - Other unintentional injuries (ICD-10 coding: W39-W39.9, W77-W77.9, W81-W81.9, W85-W87.9, X50-X58.9)
  - Self-harm (ICD-10 coding: X60-X64.9, X66-X84.9, Y87.0)
  - Inter personal injuries (ICD-10 coding: X85-Y08.9, Y87.1-Y87.2)
- Other diseases:
  - Alcohol use disorders (ICD-10 coding: E24.4, F10-F10.99, G31.2, G62.1, R78.0, X45-X45.9, X65-X65.9)
  - Epilepsy (ICD-10 coding: G40-G41.9)
  - Diabetes (ICD-10 coding: E08-E08.11, E08.3-E08.9, E10-E10.11, E10.3-E11.1, E11.3-E12.1, E12.3-E13.11, E13.3-E14.1, E14.3-E14.9)

## Table S2: comparisons of deaths March 2016-February 2017 (12 months before the taxation increase) with deaths March 2017-February 2018

|  | **Women** | **Men** | **Total** |
| --- | --- | --- | --- |
| **All-cause deaths** |  |  |  |
| Mar 2016 – Feb 2017 | 21014 | 20349 | 41363 |
| Mar 2017 – Feb 2018 | 20659 | 19100 | 39759 |
| Difference | -355 | -1249 | -1604 |
| **All CVD deaths** |  |  |  |
| Mar 2016 – Feb 2017 | 13582 | 9825 | 23407 |
| Mar 2017 – Feb 2018 | 13044 | 9176 | 22220 |
| Difference | -538 | -649 | -1187 |
